# The Editorial in the February Number

**Published:** 1894-04

**Authors:** C. Carleton Smith

**Affiliations:** Philadelphia


### THE EDITORIAL IN THE FEBRUARY NUMBER.

PHILADELPHIA, February 12th, 1894.

MY DEAR DR. JAMES:—I was much interested in your editorial in this month's HOMŒOPATHIC PHYSICIAN, in which you mention a cure of a patient under your care convalescing from an attack of "la grippe," in which Sulphur played an important part.

Sulphur is one of the most precious remedies in our materia medica, and of the first importance in clearing up obscure symptoms after the more immediate ones have been, to all intents and purposes, cured by the specifically indicated drug, besides clearing up the *débris* left in the wake of acute illnesses in the shape of latent psora. But the point I want to direct special attention to, and which your article called to mind, is the fact that there is another drug which holds a position quite as important as Sulphur in similar cases, and which must often be compared with it in order to obtain good results in the treatment thereof. I allude to Hydrastis-canad. Both Sulphur and Hydrastis have "sinking, faint feeling at pit of stomach." But a marked distinction between the two drugs consists in the fact that the latter as an accompaniment has marked palpitation of the heart. Sulphur, also, has hunger in these cases with a desire on the part of the patient to eat something to fill up the "weak, gone spot," while the Hydrastis patient has no desire to eat, no hunger, no craving for food, and if food is forced upon him the empty, gone, faint feeling is made much worse thereby.

There is another drug which must be referred to just here, viz., Lycopodium, which has an important symptom very similar to Sulphur, viz., the least portion of food causes a sense of "satiety and fullness," and hence, unless great care is taken on the part of the prescriber, the former drug may be prescribed in place of the latter, simply on the strength of this one characteristic, the result being a failure to cure. Another hint may be timely just at this point with regard to Hydrastis, a drug, by the way, which needs more careful study on the part of our brethren than it has, I fear, received. As measles is quite prevalent just now in our city, this drug will be found of the first importance in those cases which, after the skin clears off, and all other symptoms peculiar to the disease have seemingly disappeared, the child, nevertheless, does not regain its wonted health, but becomes greatly emaciated, with marked prostration and ill humor. Here Hydrastis must be studied, as it will often prove the curative remedy.

This drug is a great antipsoric, and in scrofulosis in children should never be lost sight of.

Another thought. A patient in such a condition as you describe should not be plied with stimulants. They are in no condition to digest or assimilate food. And hence they retard recovery. The simillimum first, is my motto. Then, when reaction takes place, follow with small doses of nourishment at regular intervals.

Sincerely yours in the cause of Homœopathy,

C. Carleton Smith.

